# Cost-Effective Reduced Envelope of the Stator Current via Synchronous Sampling for the Diagnosis of Rotor Asymmetries in Induction Machines Working at Very Low Slip

**DOI:** 10.3390/s19163471

**Published:** 2019-08-08

**Authors:** Jordi Burriel-Valencia, Ruben Puche-Panadero, Javier Martinez-Roman, Angel Sapena-Bano, Manuel Pineda-Sanchez

**Affiliations:** The Institute for Energy Engineering, Universitat Politècnica de València, Camino de Vera s/n, 46022 Valencia, Spain

**Keywords:** fault diagnosis, induction machines, fast Fourier transform (FFT), current envelope, Hilbert transform, Park’s vector, digital signal processor (DSP), field-programmable gate array (FPGA)

## Abstract

Fault diagnosis of rotor asymmetries of induction machines (IMs) using the stator current relies on the detection of the characteristic signatures of the fault harmonics in the current spectrum. In some scenarios, such as large induction machines running at a very low slip, or unloaded machines tested offline, this technique may fail. In these scenarios, the fault harmonics are very close to the frequency of the fundamental component, and have a low amplitude, so that they may remain undetected, buried under the fundamental’s leakage, until the damage is severe. To avoid false positives, a proven approach is to search for the fault harmonics in the current envelope, instead of the current itself, because in this case the spectrum is free from the leakage of the fundamental. Besides, the fault harmonics appear at a very low frequency. Nevertheless, building the current spectrum is costly in terms of computing complexity, as in the case of the Hilbert transform, or hardware resources, as in the need for simultaneously sampling three stator currents in the case of the extended current Park’s vector approach (EPVA). In this work, a novel method is proposed to avoid this problem. It is based on sampling a phase current just twice per current cycle, with a fixed delay with respect to its zero crossings. It is shown that the spectrum of this reduced set of current samples contains the same fault harmonics as the spectrum of the full-length current envelope, despite using a minimal amount of computing resources. The proposed approach is cost-effective, because the computational requirements for building the current envelope are reduced to less than 1% of those required by other conventional methods, in terms of storage and computing time. In this way, it can be implemented with low-cost embedded devices for on-line fault diagnosis. The proposed approach is introduced theoretically and validated experimentally, using a commercial induction motor with a broken bar under different load and supply conditions. Besides, the proposed approach has been implemented on a low-cost embedded device, which can be accessed on-line for remote fault diagnosis.

## 1. Introduction

Induction machines provide most modern industrial processes with mechanical power, as for example squirrel cage motors [1], or electrical power, as for example double-fed induction generators (DFIGs) [2]. Therefore, unexpected failures of IMs can cause heavy economic loses, extensive damages to industrial machinery, and the risk of personal hazards. Scheduled maintenance can reduce the risk of sudden breakdowns, but it must be carefully tuned to become effective. On the contrary, a continuous monitoring of the machine condition allows the detection of IM failures in an incipient stage [3] and helps limit the extension of the fault [4], so avoiding harmful, sudden stops of the production lines. Different approaches have been proposed in the technical literature for condition monitoring of IMs. Among them, the analysis of the phase currents, as in motor current signature analysis (MCSA) [5,6,7], has received in recent years an extensive research effort. It is due to its low requirements of hardware, because a simple, non-invasive current sensor is needed for monitoring the phase current, and software, because the acquired current signal is processed with a fast Fourier transform (FFT). Nevertheless, the industrial application of MCSA is challenging in some scenarios, such as large induction machines running at a very low slip, or unloaded machines tested offline, where this technique may fail, giving false positives in case of rotor asymmetries. In these scenarios, the frequencies of the fault harmonics are very close to the frequency of the fundamental, just some milihertz apart [8], and have a low amplitude, so that they may remain undetected, buried under the fundamental’s leakage until the damage is severe. To avoid false positives, some authors propose to replace the current signal by other quantities, such as the magnetic flux [9,10], vibrations [11,12,13,14,15], or modal currents [16], but these approaches require more complex hardware than MCSA. A proven approach is to use the same current acquired in MCSA, but replacing its spectral analysis by the spectral analysis of the current envelope [17], or the squared current [18].

Regarding the current envelope, the spectrum of its alternating current (AC) component displays the same fault harmonics as the original current signal, with the advantage that they appear at the true characteristic frequencies of the fault, as demonstrated in the technical literature [8,19]. Besides, the fundamental component is transformed into a direct current (DC) one, which produces no leakage in the envelope spectrum, allowing the detection of fault harmonics with a very low frequency and small magnitude. Different methods for obtaining the current envelope have been presented in the technical literature. Some of them use a single current signal, such as the modulus of the analytic signal (AS) of the current [16,20,21,22], or the Hilbert-Huang transform in [23]. Other proposal rely on the simultaneous sensing of the three-phase currents, such as the extended Park’s vector approach (EPVA) [24], based on the spectral analysis of the modulus of the current Park’s vector (PV) [25]. The EPVA has been applied to the diagnosis of stator winding faults [26], rotor faults [27], and bearing faults [28], among others.

Nevertheless, building the current spectrum using the aforementioned methods is costly in terms of computing complexity. In the case of the modulus of the analytic current signal, a Hilbert transform must be used to obtain the imaginary part of the AS of the current, before computing its modulus. On the other hand, the EPVA requires the simultaneous measurement of three-phase currents, which requires costly multi-channel simultaneous data acquisition systems. Besides, in order to avoid aliasing effects, these data acquisition systems must operate at high sampling rates (100 kHz in [29]), especially for condition monitoring of IMs fed from variable speed drives. An added difficulty, in the case of high power motors with rotor asymmetry, is the extremely low frequency of the fault harmonics, which implies a long acquisition time to achieve the required resolution (100 s in [22]). Both simultaneous requirements, a long acquisition time and a high sampling rate, generate a huge number of current samples, which increases the computing resources needed to acquire, store, and process them. Citing [30], these requirements bring an overwhelming burden to a DSP.

To use the current envelope for fault diagnosis of rotor asymmetries in an industrial environment [31], working on-line [4], in real time [32], it is necessary to reduce the hardware requirements [31,33], the sampling rate [34], and the complexity [35] of the algorithm needed to compute the current envelope; ideally, the diagnostic system should be implemented in embedded devices such as FPGAs [36], or DSPs [37], with a minimal impact on the controller tasks.

In this paper, a novel and simple approach is proposed to meet all these requirements. It consists of sampling a single phase current, synchronized with a fixed delay from its zero crossings. The number of samples that must be stored and processed with the FFT using the proposed approach is extremely low: just two samples per current’s cycle. Besides, no further processing is needed on these current values before applying the FFT, apart from taking its absolute value, which avoids the costly computation of the modulus of the analytical signal of the current, or its PV. Therefore, a very cost-effective diagnostic method with the advantages of the current envelope analysis can be built, which makes the proposed approach especially well suited for implementing the current envelope diagnostic method on low-cost, embedded field devices [38].

The outline of the paper is the following one. In Section 2, a brief explanation of the different methods for obtaining the current envelope used in fault diagnosis is presented. In Section 3 the proposed diagnostic method is developed theoretically, and in Section 4 it is validated experimentally. Besides, this method has been implemented in a low-cost embedded system, which is shown in Section 5. Finally, in Section 6 the conclusions of this work are presented.

## 2. The Current Envelope as a Fault Diagnostic Signal for Detecting Rotor Asymmetries in IMs

A rotor asymmetry fault, such as broken bars, or asymmetries in the rotor resistances, generates an amplitude modulation in the stator current, with characteristic frequencies [8]

(1) $$f_{asym}=2ksf_{1}\;k=1,2,3,\ldots$$

where $f_{1}$ is the supply frequency, and *s* is the per-unit (p.u.) rotor slip.

The fault harmonics with characteristics frequencies given by (1) can have their origin not only in bar breakages, but also in other asymmetries in the rotor windings such as cracked end rings, inherent asymmetries between bars due to tolerances and other defects during the manufacture of the cage in rotor cage machines; in the case of wound rotor machines, the fault harmonics detected in this work can be produced by high resistance joints and loss of turns in one of the phases [39].

Using (1), the expression for the current of a stator phase in an IM with a rotor asymmetry is

(2) $$i\left(t\right)=I\cos\left(2\pi f_{1}t\right)\left(1+\beta \cos(2\pi f_{asym}t)\right)$$

where *I* is the maximum value of the fundamental component of the phase current, and β is the severity of the fault, in p.u. of the fundamental component. No phase information has been included in (2) for easy of notation. In (2) it has been assumed, without any loss of generality, that the time origin coincides with a time instant when the current signal reaches its maximum value ($\cos(2\pi f_{1}t)=1$).

Considering only the main fault harmonic component, k=1 in (1), and applying trigonometrical relationships,

(3) $$i\left(t\right)=I\cos\left(2\pi f_{1}t\right)+\frac{\beta }{2}I\cos\left(2\pi f_{1}\left(1-2s\right)t\right)+\frac{\beta }{2}I\cos\left(2\pi f_{1}\left(1+2s\right)t\right)$$

where the main fault harmonics appear as side-bands of the fundamental component, at a distance $2sf_{1}$ from it. If the slip *s* is very small, as in the case of large IMs, or even in the case of small motors working under low load conditions, this distance can be very small, which may render these fault harmonics undetected, buried under the leakage of the fundamental component, until the fault is severe enough. In case of an incipient broken bar fault, for example, the value of β can be lower than β=1/200 [4].

The Fourier transform (FT) of (3) consists only of three spectral lines, at frequencies $f_{1}$, $\left(1-2s\right)f_{1}$ and $\left(1+2s\right)f_{1}$,

(4) $$\mathrm{FT}\left\{i\left(t\right)\right\}\left(f\right)=\hat{i}\left(f\right)=\frac{I}{2}\left(\delta \left(f-f_{1}\right)+\frac{\beta }{2}\delta \left(f-\left(1-2s\right)f_{1}\right)+\frac{\beta }{2}\delta \left(f-\left(1+2s\right)f_{1}\right)\right)$$

where $\hat{i}\left(f\right)$ stands for the component of the FT of i(t) located at frequency *f*, and δ is the Kronecker delta function. As the current is a real signal, only the terms of its symmetrical spectrum with positive frequency have been included in (4).

Expressing (4) in p.u. of the maximum current value, *I*, and changing to a dB scale, gives

(5) $${\hat{i}}_{dB}\left(f\right)=0\times \delta \left(f-f_{1}\right)+20\log\left(\frac{\beta }{2}\right)\times \delta \left(f-\left(1-2s\right)f_{1}\right)+20\log\left(\frac{\beta }{2}\right)\times \delta \left(f-\left(1+2s\right)f_{1}\right)$$

so that the amplitude of the main fault harmonics is

(6) $$\hat{i}\left(f_{1}+2sf_{1}\right){{|}_{dB}=\hat{i}\left(f_{1}-2sf_{1}\right)|}_{dB}=20\log\left(\frac{\beta }{2}\right)$$

Using a low value of β=1/200 in (6) gives an amplitude of the fault harmonic of only −52.04 dB, what makes it difficult to detect it in harsh industrial environments, due to the leakage of the fundamental component.

### 2.1. Current Envelope Obtained with the Modulus of the Analytic Signal of the Current

The current envelope can be obtained as the modulus of the analytic signal of the current, as has been proposed in [8,40] for the detection of broken bars failures in IMs operating at a very low slip. The AS of the stator current is defined as the complex signal

(7) $$\mathrm{AS}\left\{i\left(t\right)\right\}={\vec{i}}_{AS}=i\left(t\right)+j\cdot \mathrm{HT}\left\{i\left(t\right)\right\}$$

where AS stands for the analytic signal, and HT{i(t)} is the Hilbert transform of i(t) [8], given by

(8) $$HT\left\{i\left(t\right)\right\}=\frac{1}{\pi t}\int_{-\infty }^{\infty }\frac{i(\tau )}{t-\tau }d\tau$$

Alternatively to (8), the AS{i(t)} signal can be obtained by zeroing the negative frequencies of the spectrum of i(t), and doubling its DC value.

The AS of the stator current of an IM with rotor asymmetries (2) can be found by applying (2) to (7) [8], giving

(9) $${\vec{i}}_{AS}\left(t\right)=I\left(1+\beta \cos(2\pi \left(2sf_{1}\right)\cdot t)\right)\cdot e^{j2\pi f_{1}t}$$

The fault diagnosis procedure analyzes the modulus of (9), which is taken as the current envelope,

(10) $$|{\vec{i}}_{AS}\left(t\right)|=I\left(1+\beta \cos(2\pi \left(2sf_{1}\right)\cdot t)\right)$$

The modulus of the AS (10) contains only a DC component of value *I*, and a low frequency fault harmonic at $2sf_{1}$, with an amplitude (in dB), equal to (6), of

(11) $$\hat{|{\vec{i}}_{AS}|}\left(2sf_{1}\right){|}_{dB}=20\log\left(\frac{\beta }{2}\right)$$

### 2.2. Current Envelope Obtained with the Modulus of the Extended Park’s Vector

The current envelope can also be obtained as the modulus of the extended Park’s vector, as proposed in the EPVA. The EPVA uses the three stator currents of a three-phase IM, $i_{a}\left(t\right)$, $i_{b}\left(t\right)$ and $i_{c}\left(t\right)$, for building the current Park’s vector, defined as the complex signal [24]

(12) $${\vec{i}}_{PV}\left(t\right)=i_{D}\left(t\right)+j\cdot i_{Q}\left(t\right)$$

where

(13) $$i_{D}\left(t\right)=\frac{\sqrt{2}}{\sqrt{3}}i_{a}\left(t\right)-\frac{1}{\sqrt{6}}i_{b}\left(t\right)-\frac{1}{\sqrt{6}}i_{c}\left(t\right)$$

(14) $$i_{Q}\left(t\right)=\frac{1}{\sqrt{2}}i_{b}\left(t\right)-\frac{1}{\sqrt{2}}i_{c}\left(t\right)$$

The modulus of (12) is used for the diagnosis of rotor asymmetries in IMs. In the case of a faulty machine, with $i_{a}\left(t\right)=i\left(t\right)$ in (2), and the other two current phases forming a three-phase balanced system, it is given by

(15) $$|{\vec{i}}_{PV}\left|=\right|i_{D}\left(t\right)+j\cdot i_{Q}\left(t\right)|=\frac{\sqrt{3}}{\sqrt{2}}\cdot I\left(1+\beta \cos(2\pi \left(2sf_{1}\right)\cdot t)\right)$$

The modulus of the Park’s vector (15) is taken as the current envelope. It contains only a DC component of value $\frac{\sqrt{3}}{\sqrt{2}}\cdot I$, and a low frequency fault harmonic at $2sf_{1}$, with an amplitude (in dB), equal to (6), of

(16) $$\hat{|{\vec{i}}_{PV}|}\left(2sf_{1}\right){|}_{dB}=20\log\left(\frac{\beta }{2}\right)$$

### 2.3. Practical Issues of the Construction Current Envelope Obtained with the Modulus of the AS or with the EPVA

From (10) and (15), the current envelope has the general expression, in p.u. of its DC value, of

(17) $$i_{env}\left(t\right)=1+\beta \cos\left(2\pi (2sf_{1})\cdot t\right)$$

In a faulty machine the current envelope $i_{env}\left(t\right)$ oscillates with a frequency $f_{asym}=2sf_{1}$ (3) characteristic of the fault. Besides, its amplitude β (17) depends on the severity of the fault. Compared with the spectral analysis of the phase current, the analysis of the current envelope has three distinctive advantages, which facilitate the interpretation of the diagnostic spectrum in the search for the characteristic signatures of fault harmonics:

- The harmonic components that a rotor asymmetry fault generates are located in the current envelope spectrum at their true frequency $f_{asym}=2sf_{1}$, instead of being displayed as side-bands around the supply component, at frequencies $f_{1}\left(1\pm 2s\right)$, as can be seen in (17).
- As the frequency of these fault harmonics in (17) is generally very low, especially in the case of large IMs, the region of diagnostic interest of the current envelope spectrum is reduced to a narrow, low frequency band, compared with the diagnostic current spectrum used commonly in MCSA.
- There is no leakage produced by the fundamental component in the current envelope spectrum, because it is transformed into a DC quantity (first term of (17)).

On the other hand, the practical application of the current envelope for the diagnosis of rotor asymmetries is problematic in case of large motors, with a very low rated slip. In this case, the frequencies of the fault harmonics (3) are very close to the supply frequency (*f*_1_), which requires sampling the currents during a long acquisition time (*t_acq_*) to achieve enough frequency resolution in the spectrum of the current envelope. A high sampling frequency (*f_s_*), combined with a long sampling time (100 kHz, 100 s in [22]), implies that a huge number of current samples must be acquired, stored, and processed, what hinders the implementation of the current envelope diagnostic method using low-cost field devices with limited resources. Besides, both methods of computing the current envelope have different additional drawbacks:

- The AS method implies the computation of the Hilbert transform (8), which involves all the current samples, or the use of a Hilbert filter to generate it on-line.
- The EPVA requires sampling simultaneously three stator currents for obtaining the Park’s vector modulus (PVM) (12) which, at high sampling rates $f_{s}$, requires a costly multi-channel simultaneous data acquisition systems, and increases the computing resources by a factor of three, compared to the analysis of a single current signal.
- In both methods, the modulus of a complex signal, (10) of (15), must be computed prior to process it with the FFT.

## 3. Proposed Methodology: Reduced Current Envelope Obtained via Synchronized Sampling of the Current

The proposed method maintains the advantages of the use of the current envelope, while avoiding the drawbacks of the AS method or the EPVA. It is based on a particular feature of the fault diagnosis of IMs based on the analysis of the current envelope, namely that its fault components (17) have a very low frequency. In the case of a rotor asymmetry, for example, and taking into account that the slip *s* is small (it varies typically between 0% to 10%), the frequency of the induced fault harmonics (1) is also small (in the range of 0% to 20% of the frequency of the fundamental component, $f_{1}$ [41]). Therefore, the current envelope obtained with (10) or (15) is usually decimated before generating the diagnostic spectrogram, using the FFT. Instead, the proposed method is based on the synchronous sampling of just one phase current twice per period of the fundamental component, of frequency $f_{1}$. This reduced set of current samples constitutes the proposed reduced current envelope signal, which contains the same information about the fault harmonics as the full-length current envelope, but at a fraction of its acquisition, storage and computing costs.

The process of sampling the phase current of a faulty machine (9) consists of acquiring current samples during an acquisition time $t_{acq}$, with a sampling frequency $f_{s}$. This gives a sequence of $N=f_{s}\cdot t_{acq}$ current samples i[k]

(18) $$i\left[k\right]=I\cos\left(2\pi f_{1}t_{k}\right)\left(1+\beta \cos(2\pi f_{asym}t_{k})\right)$$

where

(19) $$t_{k}=k\Delta t=\frac{k}{f_{s}}\;\;\;k=0,1,\ldots ,N-1$$

The method proposed in this paper for obtaining the new diagnostic signal, the reduced current envelope $i_{renv}\left[k\right]$, consists of:

1. Performing the sampling process at a rate equal to twice the fundamental frequency, i.e., is $f_{s}=2f_{1}$ in (19). This is achieved by synchronizing the sampling process with the zero crossings of the current.
2. Shifting the sampling process a fixed time delay from the zero crossings of the current.
3. Taking the absolute value of the resulting set of samples.

In this way, the time instants used for sampling the current are given by

(20) $$t_{k}^{\prime }=t_{0}+k\Delta t^{\prime }=t_{0}+\frac{k}{2f_{1}}\;\;\;k=0,1,\ldots N-1$$

Applying (20) to (18), and taking absolute values, gives the reduced current envelope as

(21) $$i_{renv}\left[k\right]=I|\cos\left(2\pi f_{1}t_{k}^{\prime }\right)|\left(1+\beta \cos(2\pi f_{asym}t_{k}^{\prime })\right)$$

that is,

(22) $$i_{renv}\left[k\right]=I|\cos\left(2\pi f_{1}t_{0}+k\pi \right)|(1+\beta \cos(2\pi \left(2sf_{1}\right)\left(t_{0}+\frac{k}{2f_{1}}\right))$$

The spectrum of this new signal $i_{renv}\left[k\right]$ contains only a DC component of value $I|\cos(2\pi f_{1}t_{0})|$, and a low frequency fault harmonic at $f_{asym}=2sf_{1}$, with an amplitude, in dB scale (after normalizing it to p.u. values of the DC component) of

(23) $$\hat{i_{renv}}\left(2sf_{1}\right){|}_{dB}=20\log\left(\frac{\beta }{2}\right)$$

which coincides exactly with the value obtained with the AS method (10) and with the EPVA (15).

As for the choice of the delay time from the zero crossings, in step 2, the optimal choice would be to maximize the DC component $I|\cos(2\pi f_{1}t_{0})|$ in (22), in order to achieve the maximum signal-to-noise ratio. This implies having $2\pi f_{1}t_{0}=0$, i.e., sampling the current when the fundamental component reaches its maximum value, which is delayed an angle π/2 from its zero crossings. This choice maximizes the amplitude of $i_{renv}\left[k\right]$, as in [22]. A delay angle different than π/2 (or 3π/2) can also be used in (22), but the signal-to-noise ratio of the sampled current would be lower.

The advantages of using the reduced current envelope $i_{renv}\left[k\right]$, compared with the AS or the EPVA, are the following ones:

- Instead of using a multi-channel simultaneous data acquisition system for sampling simultaneously the three stator currents at a high *f_s_* rate, as in the case of the EPVA, just a low-speed analog-to-digital converter (ADC) is needed for sampling a single phase current.
- The number of current samples that are needed for building the current envelope is reduced from $f_{s}\times t_{acq}$ to only $2f_{1}\times t_{acq}$ in (22), which represents a minimal fraction $\frac{2f_{1}}{f_{s}}$ of the full-length AS or PVM samples.
- The computation burden of obtaining the modulus of AS (10) or PVM (15) is eliminated, because the current samples need no post-processing treatment before applying the FFT, apart from taking its absolute value.
- The number of operations needed for generating the spectrum of the current envelope is reduced in the same proportion as the reduction in the number of current samples.

### Practical Implementation of the Proposed Methodology

**Figure 1:**
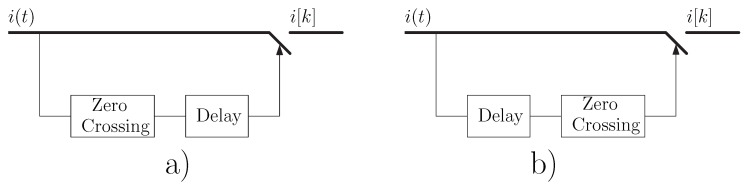
Practical algorithms to compute the proposed diagnostic signal $i_{renv}\left[k\right]=|i\left[k\right]|$. In (**a**) the positive zero crossings of the current are first detected, and the sampling process is delayed from these time instants. In (**b**) the current signal is first delayed, and the sampling process is synchronized with the positive zero crossings of the delayed signal.

From a practical point of view, the proposed synchronized sampling of the phase current can be implemented using two variants, presented in Figure 1:

(a) The zero crossings of the phase current are first detected, and the sampling process is carried on after a fixed delay with respect to these time instants (Figure 1a). This delay can be implemented using a timer delay unit, triggered by the zero-crossing detector.
(b) The phase current is first delayed, and the sampling process is carried on after a fixed delay with respect to the zero crossings of the delayed current signal (Figure 1b). This approach has been used in [22], using a Hilbert filter as delay unit.

**Figure 2:**
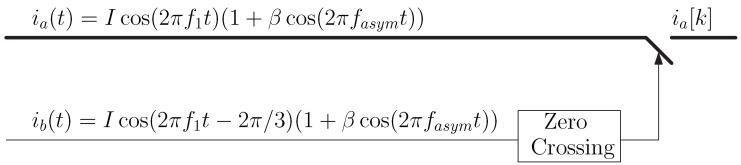
Simplified algorithm to obtain the proposed diagnostic signal $i_{renv}\left[k\right]=|i_{a}\left[k\right]|$ in three-phase systems, eliminating the delay unit of Figure 1b. It consists of synchronizing the sampling instants of one of the phase currents of the faulty machine, $i_{a}\left(t\right)$, with the zero crossings of one of the other phase currents, $i_{b}\left(t\right)$, and taking its absolute value.

A further simplification can be achieved with the elimination of the delay component in Figure 1b. In case of three-phase IMs, a phase current different than the one that is being sampled has a fixed delay of ±2π/3. Therefore, the synchronization sampling of a given current $i_{a}\left(t\right)$ can be carried using the zero crossings of one of the other phase currents $i_{b}\left(t\right)$, without needing any delay unit, as seen in Figure 2. This approach has been used previously in a different context in [38], for building the reduced Park’s vector modulus, and it is the alternative that will be used in the experimental part of this work. It is worth mentioning that the method presented in [38] can be considered, under this new point of view, a particular implementation of the much more general approach proposed in this work.

## 4. Experimental Validation

**Figure 3:**
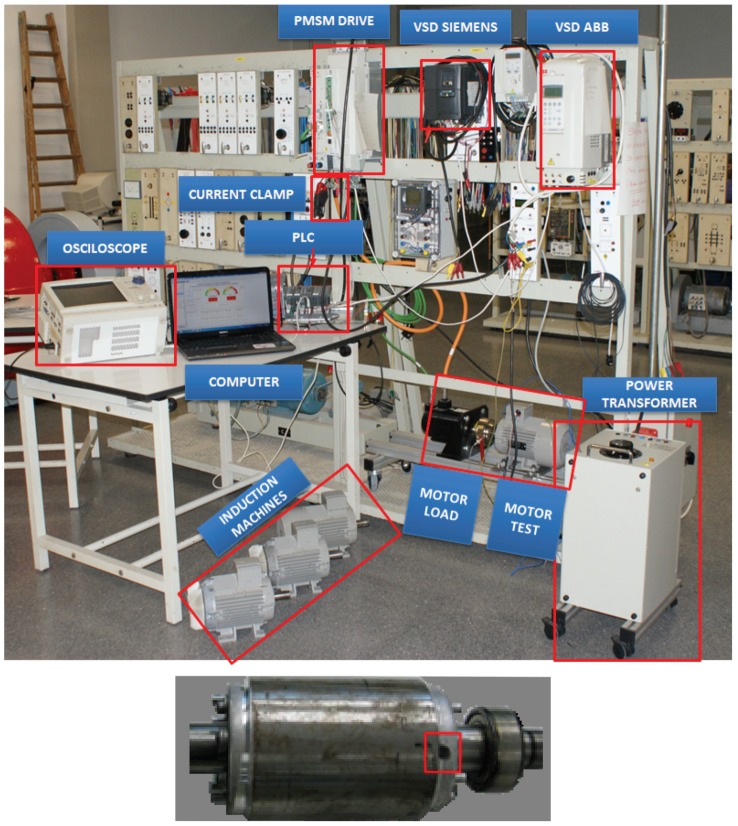
Top: test bench used for the experimental tests presented in this work. Bottom: detail of the rotor asymmetry provoked by drilling a hole in one of the rotor bars.

The proposed method has been validated using a commercial squirrel IM (see Appendix A) with a broken bar failure, provoked by drilling a hole in one of the rotor bars, as seen in Figure 3, bottom. To assess the validity of the proposed method under a wide range of industry working conditions, this motor has been tested under different supply and load levels, using the test bench shown in Figure 3, top. The following parameters can be adjusted using the test bench:

**Supply**  Direct connection to the mains or connection through one of the two different variable speed drives (VSDs) available in the bench (ABB ACS800-01-0005-3+E200+L503, or Siemens Micromaster 440). Besides, the VSDs can be operated with different control methods, Volts per Hertz (V/Hz) or sensorless vector control (SVC).
    **Load**  The motor load can be controlled using a permanent magnet synchronous machine (PMSM) (see Appendix A, motor type II), connected to its shaft, driven by servo driver (ABB ACSM1-04AS-024A-4+L516), which provides an accurate control of the load torque.

The characteristics of the current probe used for data acquisition are given in Appendix B. The circuit used for the detection of the zero crossings of the current (represented as the zero-crossing box in Figure 2) is the circuit presented in [42].

**Figure 4:**
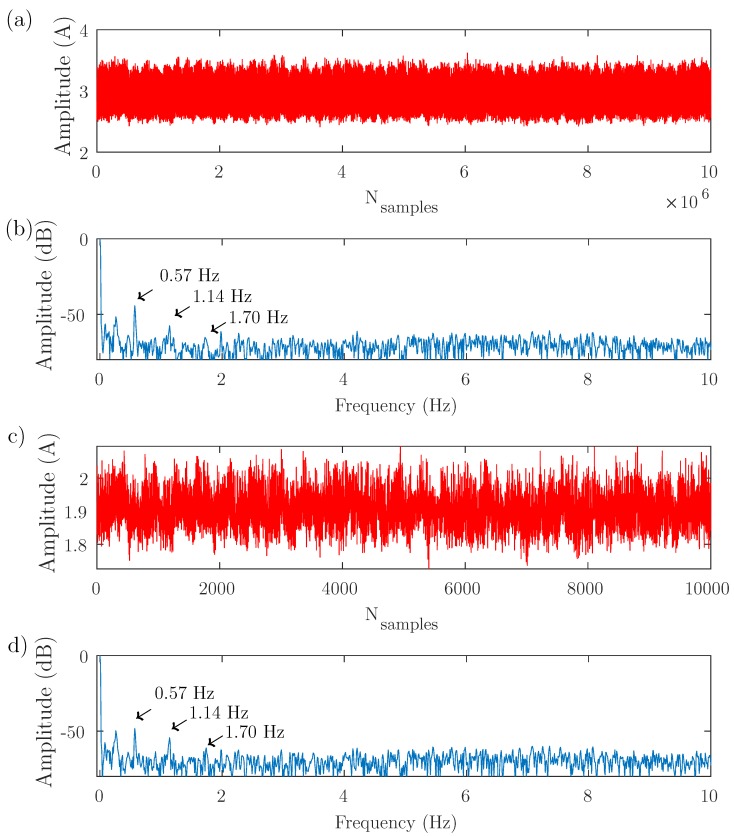
Test 1. Motor fed through the ABB VSD in V/Hz control mode, a reference speed of 3000 r/min, and without any load (speed = 2983 r/min, slip = 0.0057 p.u.). (**a**) Full-length current envelope ($10^{7}$ samples) and its spectrum (**b**), showing the three first fault harmonics. (**c**) Reduced current envelope (10,000 samples), and its spectrum (**d**). Both spectra show the same fault harmonics, although the reduced current envelope contains only the 0.1% of the original number of samples.

**Figure 5:**
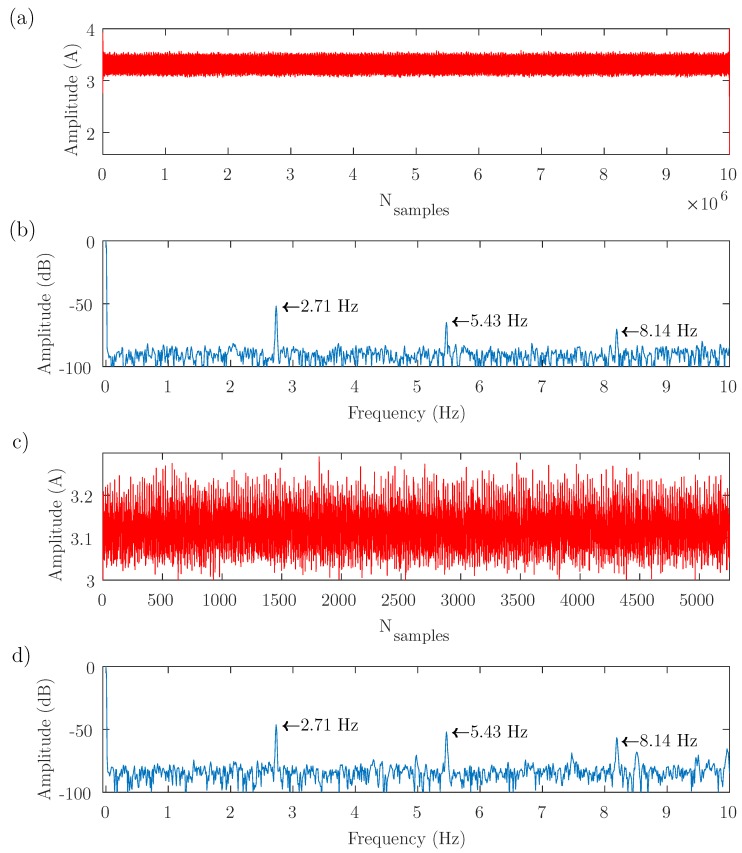
Test 2. Motor fed through the Siemens VSD in SVC mode, a reference speed of 1530 r/min, and a load equal to 35% of its rated load (frequency output = 26.22 Hz, speed = 1530 r/min, slip = 0.0271 p.u.). (**a**) Full-length current envelope ($10^{7}$ samples) and its spectrum (**b**), showing the three first fault harmonics. (**c**) Reduced current envelope (5244 samples), and its spectrum (**d**). The same fault harmonics appear in both spectra, although the reduced current envelope contains only the 0.05% of the original number of samples.

**Figure 6:**
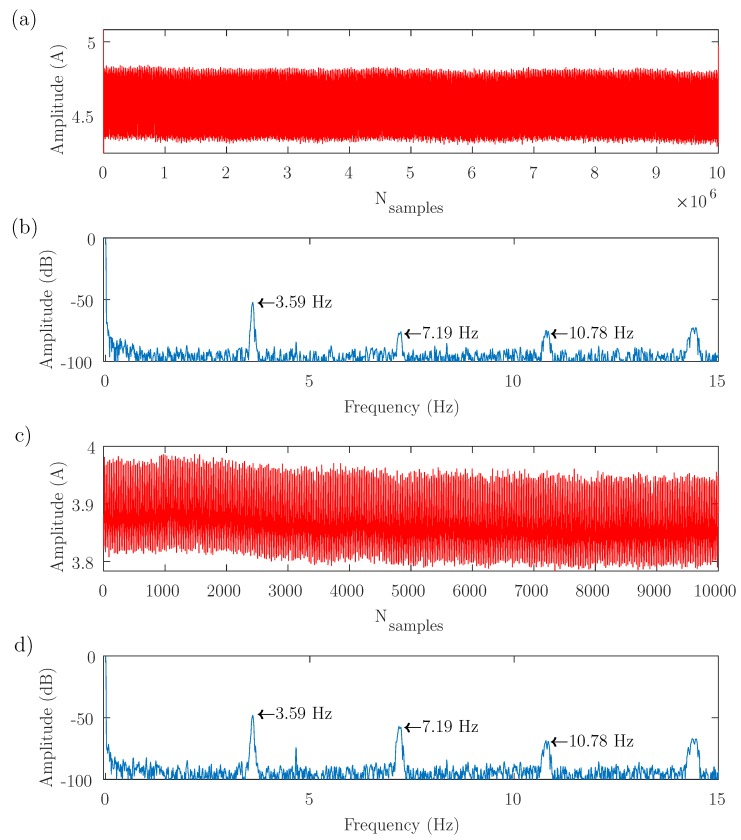
Test 3. Motor fed directly from the mains, and rated load (speed = 2892 r/min slip = 0.0359 p.u.). (**a**) Full-length current envelope ($10^{7}$ samples) and its spectrum (**b**), showing the three first fault harmonics. (**c**) Reduced current envelope (10,000 samples), and its spectrum (**d**). Both spectra show the same fault harmonics, although the reduced current envelope contains only the 0.1% of the original number of samples.

The test bench depicted in Figure 3 has been used to perform three experimental tests, with different supply, control, and load conditions, as presented in Table 1. The results obtained with these tests are shown in Figure 4, Figure 5 and Figure 6, respectively. In each of these figures, the full-length current envelope (a) and its power spectrum (b) are compared with the proposed reduced current envelope (c) and its power spectrum (d). Besides, the expected frequencies of the fault harmonics, given by (3), are marked with arrow labels.

For validation purposes, the experimental results obtained with the proposed approach are compared with the results obtained with other methods, the modulus of the AS of one of the phase currents (10), and the PVM (15). For building the full-length current envelope using both methods, the three-phase currents have been sampled during 100 seconds at a rate of $f_{s}=100$ kHz, giving a total amount of $10^{7}$ samples of the current envelope.

Figure 4 shows the first test, with the motor fed through the ABB VSD in V/Hz control mode, a reference speed of 3000 r/min, and without any load (speed = 2983 r/min, slip = 0.0057 p.u.). The full-length current envelope has been computed using the PVM method (15).

Figure 5 shows the second test, with the motor fed through the Siemens VSD in SVC mode, a reference speed of 1530 r/min, and a load equal to 35% of its rated load (frequency output = 26.22 Hz, speed = 1530 r/min, slip = 0.0271 p.u.). In this case, the full-length current envelope has been computed using the AS method (10).

Finally, Figure 6 shows the third test, with the motor fed directly from the mains, and rated load (speed = 2892 r/min slip = 0.0359 p.u.). In this case, again, the full-length current envelope has been computed using the AS method (10).

As demonstrated theoretically, it can be seen in Figure 4, Figure 5 and Figure 6 that the same diagnostic information is displayed in the power spectrum of the full-length current envelope and in the power spectrum of the reduced current. But the proposed approach can obtain these results with a small fraction of the storage and computing resources required by the traditional methods (several orders of magnitude lower). This reduction has been quantified in Table 2 (number of current samples) and in Table 3 (computing time used to obtain the power spectrum). All these comparisons have been done using a personal computer, whose characteristics are given in Appendix C.

## 5. Implementation on a Low-Cost Embedded System

**Figure 7:**
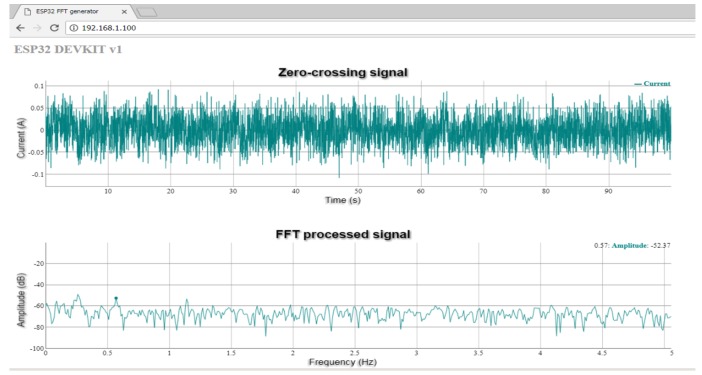
Reduced current envelope (**top**) and its spectrum (**bottom**) for the motor current in Test 1, generated in the built-in web server of the ESP32 device with the proposed approach.

To validate the advantages of the proposed approach for building low-cost diagnostics systems, it has been implemented on a low-power ESP32 device from Espressif Systems, whose main characteristics are given in Appendix D. This on-line system acquires the current of phase *B* of the induction motor when the current in phase *A* crosses the zero level. A 8192-points FFT implemented on the device obtains the spectrum of the sampled current. This spectrum can be accessed on-line via the device’s built-in web server, using a web browser, as depicted in Figure 7.

**Figure 8:**
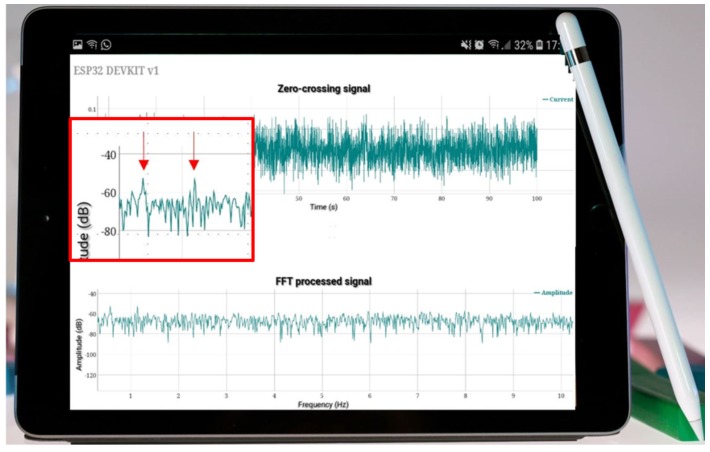
Diagnostic spectrum generated with the proposed approach, displayed on a portable device for easy access by maintenance personnel.

Figure 8 shows the diagnostic spectrum displayed on a portable device for easy access by the maintenance personnel.

## 6. Conclusions

In this paper, a novel procedure for building a reduced current envelope, using a low frequency synchronized sampling of the phase current, has been presented. The proposed approach is a cost-effective method for implementing the fault diagnosis of rotor asymmetries in IMs, using on-line devices with limited resources. It has been demonstrated theoretically that the reduced current envelope obtained in this way contains the same fault harmonics as the full-length current envelope signal, but using a small fraction of storage and computing resources. The proposed approach has been validated experimentally using a 1.5 kW commercial motor with a broken bar, tested under different supply and load working conditions.

As for the practical implementations of the synchronizing procedure for obtaining the reduced current envelope, a work in progress is the development of fast and simple algorithms to implement this process using a phase lock loop (PLL), and to apply it to the fault detection of other types of faults.

## Figures and Tables

**Table 1:** Experimental tests.

N.	Connection	Control	Frequency (Hz)	Load
1	ABB VSD	V/Hz	50	Unloaded
2	Siemens VSD	SVC	26.22	35% of rated load
3	direct on line (DOL)		50	Rated load

**Table 2:** Comparison between the number of current samples of the full-length current envelope and of the reduced current envelope proposed in this work.

Test	Full Length	Reduced Length	Reduction Ratio (%)
	Current Envelope	Current Envelope	
**N°**	**(A)**	**(B)**	$\frac{\left(B\right)}{\left(A\right)}\cdot 100$
1	$10^{7}$ samples	10,000 samples	0.1%
2	$10^{7}$ samples	5244 samples	0.05%
3	$10^{7}$ samples	10,000 samples	0.1%

**Table 3:** Comparison between the time (seconds) needed to compute the power spectrum of the full-length current envelope and of the reduced current envelope proposed in this work.

Test	Full Length	Reduced Length	Reduction Ratio (%)
	Current Envelope	Current Envelope	
1	1.32 s	0.01 s	0.76%
2	1.33 s	0.009 s	0.68%
3	1.33 s	0.01 s	0.75%

## Appendix A. Motors Characteristics

Motor type I: SIEMENS 1LA7090-2AA10. Three-phase induction motor, P=1.5 kW, f=50 Hz, U=400 V, I=3.3 A, n=2860 r/min, and cosφ=0.85.

Motor type II: ABB MS4839N4008E43C10. Permanent magnet synchronous machine, P=4.9 kW, f=50 Hz, U=400 V, T=15.5 Nm, I=14.4 A, and n=3000 r/min.

## Appendix B. Current Clamp Characteristics

CHAUVIN ARNOUX model MN 60. Current range: 0.1 A AC … 20 A AC (60 A peak). Output signal: 100 mV AC/A AC (2 V for 20 A). % Accuracy of output signal ≤2% + 50 mV. Bandwidth 40 kHz. 600 V category III, pollution degree 2 (IEC-1010-1). Transformation rate 1A = 10 mV.

## Appendix C. Computing Platform

Intel Core i7-2600K CPU @ 3.40 GHZ, 16 GB RAM, with MATLAB Version: 9.6.0.1072779 R2019a.

## Appendix D. Esp32 Embedded System

ESP32 2.4 GHz WiFi and Bluetooth combo chip designed with the TSMC ultra low power. ESP32 contains two low-power Xtensa${}^{®}$ 32-bit LX6 microprocessors. Programming environment: IDE Arduino with ESP32 API. ADC: MCP3008-I/P 10-bit SPI, 8 input channels, industrial temperature, PDIP package.
